# Emergence of BA9 genotype of human respiratory syncytial virus subgroup B in China from 2006 to 2014

**DOI:** 10.1038/s41598-017-17055-0

**Published:** 2017-12-01

**Authors:** Jinhua Song, Huiling Wang, Jing Shi, Aili Cui, Yanzhi Huang, Liwei Sun, Xingyu Xiang, Chaofeng Ma, Pengbo Yu, Zifeng Yang, Qi Li, Teresa I. Ng, Yan Zhang, Rongbo Zhang, Wenbo Xu

**Affiliations:** 1 WHO WPRO Regional Reference Measles/Rubella Laboratory and Key Laboratory of Medical Virology, Ministry of Health, National Institute for Viral Disease Control and Prevention, China Center for Disease Control and Prevention, Beijing, People’s Republic of China; 2 Lu Juan Community Health Center of Daxing region, Beijing, People’s Republic of China; 3 Jilin Children’s Medical Center, Children’s Hospital of Changchun, Changchun, People’s Republic of China; 4 Hunan Provincial Centers for Disease Control and Prevention, Changsha, People’s Republic of China; 5 Xian Center for Disease Control and Prevention, Xian, People’s Republic of China; 6 Shaanxi Provincial Centers for Disease Control and Prevention, Xian, People’s Republic of China; 7 State Key Laboratory of Respiratory Disease, National Clinical Research Center for Respiratory Disease, First Affiliated Hospital of Guangzhou Medical University, Guangzhou, People’s Republic of China; 8 Hebei Provincial Centers for Disease Control and Prevention, Shijiazhuang, People’s Republic of China; 9 AbbVie, Inc, North Chicago, IL USA; 10 Medical College, Anhui University of Science & Technology, Huainan, People’s Republic of China

## Abstract

A study was conducted to investigate the circulation of HRSV subgroup B (HRSVB) in China in recent years. HRSVB sequences from 365 samples collected in 1991, 2004 and 2008–2014 in China, together with 332 Chinese HRSVB sequences obtained from GenBank were analyzed to determine the geographic and yearly distribution of HRSVB. Phylogenetic analysis revealed these HRSVB sequences clustered into 4 genotypes with different frequencies: BA (83%), CB1 (11%), SAB (3.0%) and GB3 (0.7%). Between 2005 and 2013, there was a co-circulation of BA and non-BA genotypes in China. Genotypes BA9 and BA10 were two of the main BA genotypes detected in this study. Genotype BA9 was first detected in China in 2006 and became the predominant HRSVB genotype circulating in China from 2008 to 2014. Three different lineages were detected for both genotypes BA9 and BA10. Time to the most recent common ancestor for genotypes BA9 and BA10 was estimated for years 1997 and 1996, respectively. Results of this study not only contribute to the understanding of the circulation pattern, but also the phylogenetic pattern and evolution of HRSVB in China from 1991 to 2014.

## Introduction

Human respiratory syncytial virus (HRSV) is the major cause of acute lower respiratory tract infection (ALRTI) worldwide in infants and young children (<5 years of age), as well as in the elderly and patients who are immunocompromised^1–3^. In China, among 28,369 patients with ALRTI from 81 sentinel hospitals in 22 provinces, HRSV was the most frequently detected virus (9.9%) and was also the most common etiology of ALRTI in children <2 years of age in China^4–6^.

HRSV is a member of the *Paramyxoviridae* family and the *Pneumovirinae* subfamily. The HRSV virion consists of a non-segmented, single-strand negative RNA genome packaged in a lipid envelope. The genome of HRSV is about 15.2 kb in length and encodes 11 proteins: NS1, NS2, N, P, M, SH, G, F, M2-1, M2-2, and L. The F and G proteins are the most important viral transmembrane surface glycoproteins. The F protein is highly conserved and the G protein is highly variable. According to the reactivity with monoclonal antibodies against surface glycoproteins, HRSV strains have been classified as subgroup A (HRSVA) or subgroup B (HRSVB)^7^. To date, based on the sequence of the second hypervariable region (HVR2) of the G protein, HRSVA has been divided into 14 genotypes, GA1-GA7^8^, SAA1^9^, CB-A^10^, NA1-4^11^ and ON1^12^, whereas HRSVB has been divided into 24 genotypes, GB1-GB4^8^, SAB1-SAB4^13^, URU1-2^14^, BA1-12^10,15,16^, CB1^11^, GB5^17^, and CBB^10^.

In a previous study, we found that HRSVB predominated between 2008 and 2010 in China while HRSVA predominated in 2010 and 2012, and both subgroups co-circulated in China till March 2014^18^. We also reported that the ON1 genotype became the predominant genotype of HRSVA circulating in China between 2013 and 2015. In this report, we describe the molecular epidemiology, circulation pattern, and evolution of different HRSVB genotypes in 6 representative regions of China from 1991 to 2014. One of the HRSVB genotypes circulating worldwide in this period of time was the BA genotype, which was first detected with a 60-nucleotide duplication in the C-terminal of the G gene in 1998. Since then, the BA genotype has spread worldwide, and 12 genotypes of BA have been identified^10,15,16,19,20^. While the BA genotype has been reported circulating in different local regions in China including Gansu^21^, Beijing^11^, Chongqing^17,22^, Shanghai^23^, Zhejiang^24^ and Sichuan^25^ during the epidemic seasons from 2006 to 2013, the epidemiology of all HRSVB genotypes in China during this period time has not been studied extensively. In this study, the circulation and evolution of Chinese HRSVB genotypes were analyzed and this study represents one of the biggest and most extensive studies to investigate the circulation of HRSVB in different parts of China in the past decade.

## Results

### Samples and sequences information

4115 samples were collected from patients diagnosed with respiratory infection from 5 representative regions of China from 2008 to 2014. A total of 656 out of these 4115 (15%) samples were identified as positive for HRSV. Of these, 295 samples were identified as HRSVA and 360 were identified as HRSVB, and 1 sample was identified to be positive for both HRSVA and HRSVB. Analysis of the HRSVA sequences has been reported previously^18^. In this study, 361 HRSVB sequences, together with sequences from 2 HRSVB samples collected from Jilin in 1991, as well as sequences from 2 HRSVB samples collected from Beijing in 2004, were analyzed.

In addition, 332 Chinese HRSVB sequences were downloaded from GenBank. Therefore, a total of 697 sequences from 13 provinces/cities of 6 geographically distinct regions of China were analyzed to determine the molecular epidemiology of HRSVB in China. Among these 697 sequences, 100 of them were obtained from the Dongbei region, 252 were obtained from the Huabei region, 76 were collected from the Huadong region, 71 were collected from the Xibei region, 95 were obtained from the Xinan region, and 103 were collected from the Zhongnan region (Table 1 and Fig. 1).

**Table 1:** Distribution of HRSVB samples collected in China from 1991 to 2014.

Region	Dongbei	Huabei		Xibei		Xinan		Zhongnan			Huadong			Total
Province/City	Jilin	Beijing	Hebei	Shaanxi	Gansu	Sichuan	Chongqing	Hong Kong	Guangdong	Hunan	Zhejiang	Shandong	Shanghai	
1991	2(2)	—	—	—	—	—	—	—	—	—	—	—	—	2(2)
2000	—	—	—	—	—	—	—	3(0)	—	—	—	—	—	3(0)
2001	—	—	—	—	—	—	—	3(0)	—	—	—	—	—	3(0)
2004	—	4(2)	—	—	—	—	—	4(0)	—	—	—	—	—	8(2)
2005	—	5(0)	—	—	—	—	—	6(0)	—	—	—	—	—	11(0)
2006	—	—	—	—	—	—	4(0)	3(0)	—	—	—	—	—	7(0)
2007	—	3(0)	—	—	—	—	1(0)	1(0)	—	—	—	—	—	5(0)
2008	—	16(11)	—	—	28(0)	—	15(0)	2(0)	—	—	—	—	—	61(11)
2009	—	144(107)	—	—	1(1)	—	11(0)	5(0)	2(2)	—	—	—	16(0)	179(110)
2010	—	48(26)	1(1)	2(2)	2(2)	8(0)	5(0)	9(0)	9(9)	—	—	—	27(0)	111(40)
2011	—	7(0)	—	—	—	6(0)	6(0)	—	1(1)	—	—	—	1(0)	21(1)
2012	2(2)	5(0)	—	—	—	4(0)	2(0)	—	2(0)	11(11)	—	—	2(0)	28(13)
2013	34(34)	—	3(3)	30(30)	—	22(0)		—	5(0)	13(13)	28(0)	—	—	135(80)
2014	62(62)	15(15)	1(1)	8(8)	—	11(0)	—	—	—	24(18)	—	2(2)	—	123(106)
Total	100(100)	247(161)	5(5)	40(40)	31(3)	51(0)	44(0)	36(0)	19(12)	48(42)	28(0)	2(2)	46(0)	697(365)

Numbers shown are the sum of sequences collected in this study and those downloaded from GenBank; sequences collected in this study are shown within parentheses. The 6 representatives regions of China in our study were Dongbei (Jilin), Huabei (Beijing and Hebei), Xibei (Shaanxi and Gansu), Xinan (Sichuan and Chongqing), Zhongnan (Guangdong, Hunan and Hong Kong SAR), and Huadong (Shandong, Shanghai and Zhejiang).

**Figiure 1:**
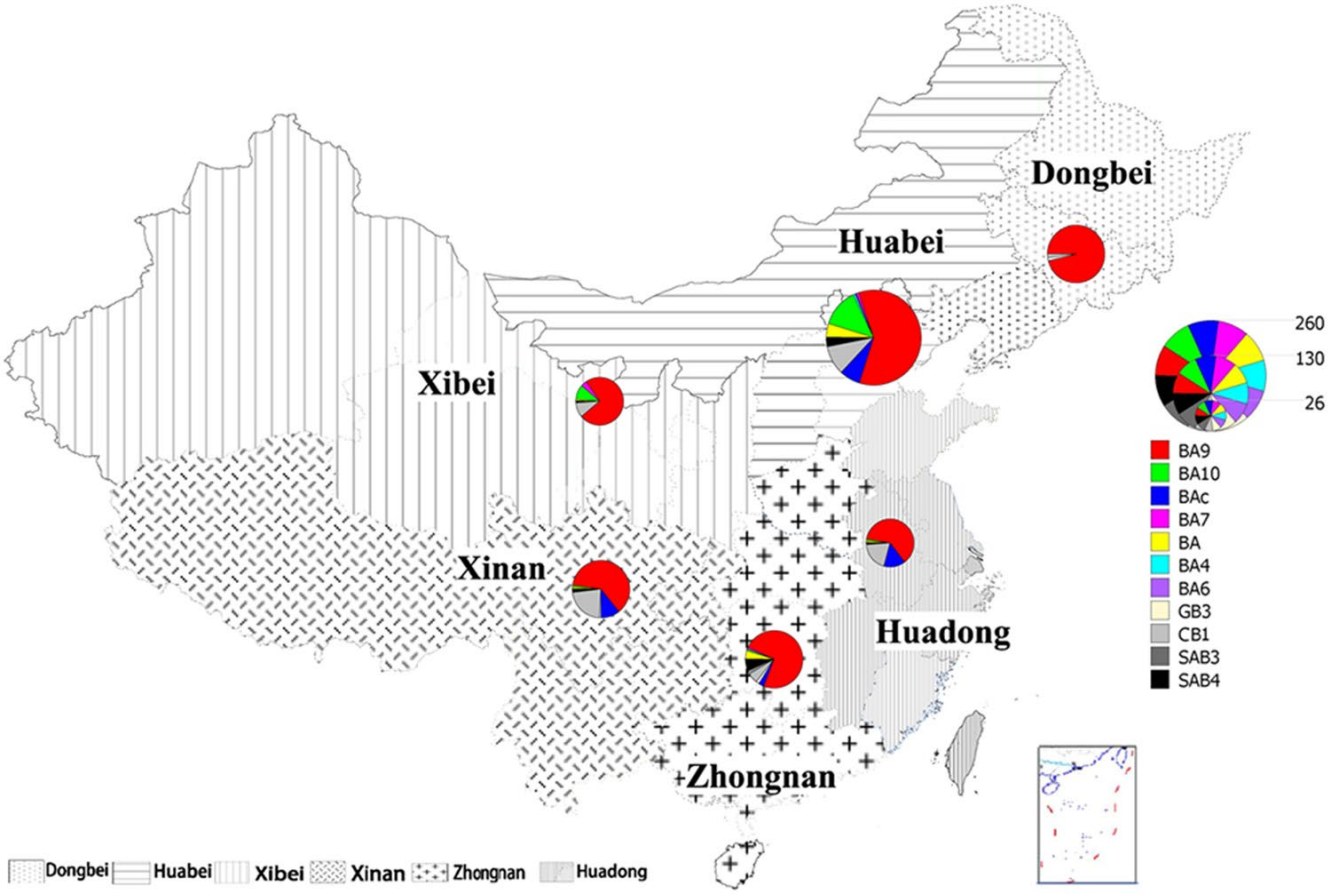
Geographic distribution of HRSVB samples in 6 representative regions of China. The region and the number of sequences are colored according to the legend. The map was constructed using Mapinfo Professional software, Version 11.0 (http://www.pitneybowes.com/us/location-intelligence/geographic-information-systems/mapinfo-pro.html).

### Clinical characteristic of HRSVB infection

Clinical information was available for 317 out of the 365 patients from whom the HRSVB samples were collected in this study. Most (92%, n = 292) of the 317 samples were collected from inpatients and 8% (n = 25) were from outpatients. The ages of the HRSVB-infected patients ranged from 1 day to 68 years old; the majority (97%) of the patients were infants or children younger than 5 years of age, including 127 (40%) females and 190 (60%) males. For the clinical diagnoses, bronchitis with pneumonia was the leading diagnosis with 158 (50%) cases, 104 (33%) were diagnosed with pneumonia, 7% were diagnosed with upper respiratory tract infection, and 2% were diagnosed with other diseases (1 case with appendicitis, 1 case with enteritis and 3 cases with unknown diagnoses). Complications occurred mainly in children under 2 years of age, with 12% (39/317) having one or more complications. The most common complications were heart disease 38% (15/39) and respiratory failure 15% (6/39). Other complications included thrush, diarrhea, asthma, biliary atresia and atelectasis.

### Phylogenetic analysis of Chinese HRSVB sequences

**Figure 2:**
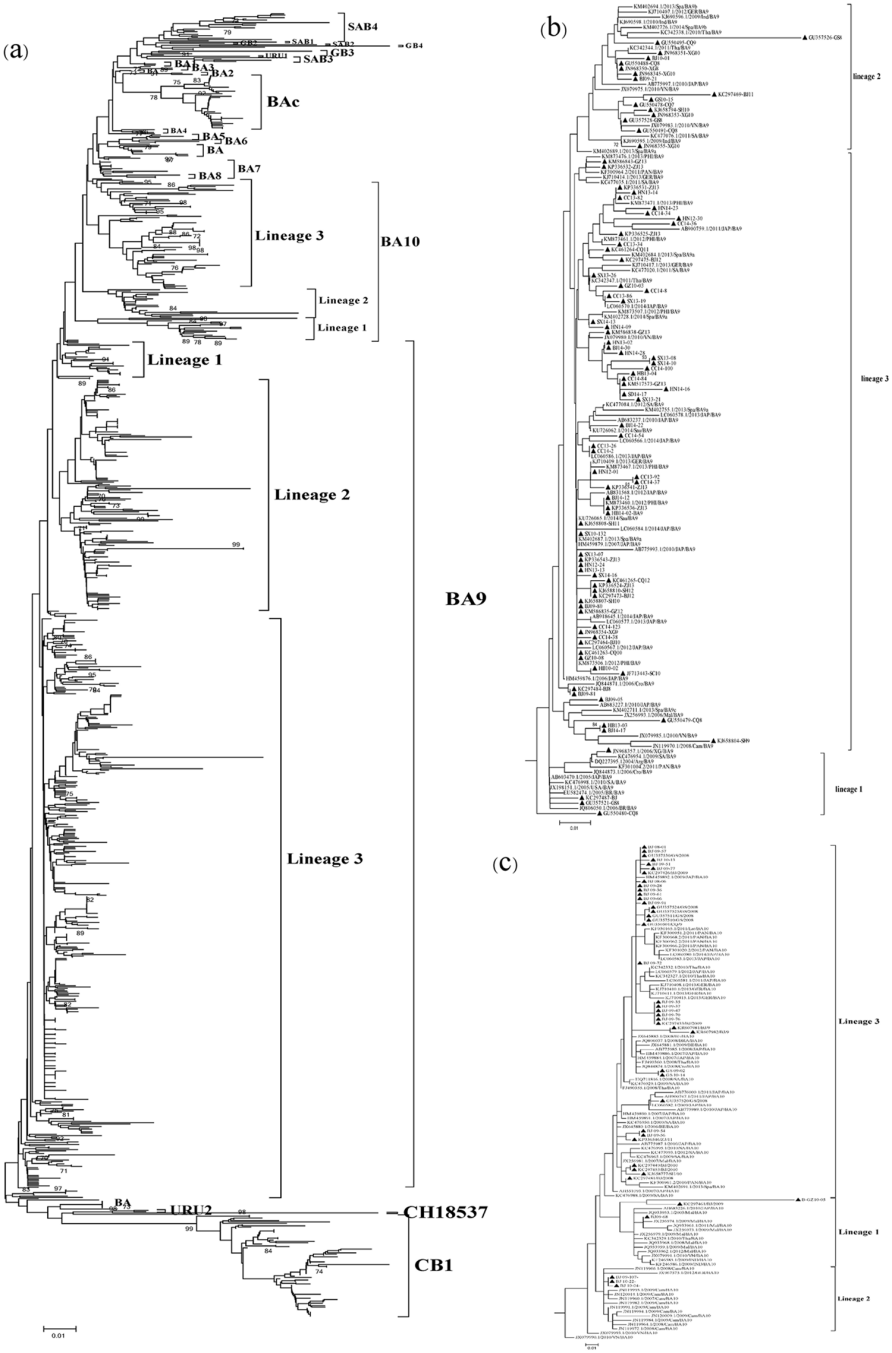
(**a**) Phylogenetic tree of 697 Chinese HRSVB sequences from 1991 to 2014 with reference sequences from Genbank. (**b**) Phylogenetic tree of 92 BA9 sequences from China and 64 BA9 reference sequences from other countries. (**c**) Phylogenetic tree of 43 BA10 sequences from China and 71 BA10 reference sequences from other countries. Chinese sequences were denoted by triangles in Fig. 2b,c. A detailed tree of Fig. 2a is shown in Supplementary Figure 1.

All of the HRSVB sequences were classified into 4 main genotypes (Fig. 2a and Supplementary Figure 1). Most (83%) of the sequences belonged to the BA (BA9, BAc, BA10, BA, BA7, BA4, and BA6) genotype, followed by the CB1 genotype, SAB (SAB3 and SAB4) genotype and GB3 genotype with prevalence of 11%, 3.0%, and 0.7%, respectively. Genotypes BA9, BAc and BA10 were the main clusters of BA genotype with 68%, 6.2% and 6.2% in overall prevalence. The BA9 genotype, the largest cluster in this study, included sequences from 13 provinces from 2006 to 2014. The BA10 genotype clustered mostly with sequences collected from samples from Gansu in 2008 and in Beijing from 2009 to 2010. Three different lineages were detected for the BA9 as well as the BA10 genotypes (Fig. 2a and Supplementary Figure 1).

Forty-two Chinese BAc sequences were collected from 5 regions of China (all 6 regions except the Dongbei region) between 2008 to 2010, which constituted an independent lineage with 78% bootstrap value (Fig. 2a and Supplementary Figure 1). A total of 76 sequences from 6 regions clustered with the sequence of genotype CB1 (7 of these 76 sequences were designated as genotype GB5 in a previous publication^17^) and this genotype circulated in China between 2008 and 2013. HRSVB SAB3 strains were only found in Hong Kong SAR in 2000 and 2004, while SAB4 strains circulated in all regions except the Dongbei region during 2000–2001 and 2004–2010 seasons after this genotype was first detected in Jilin. Only 2 sequences from Hong Kong SAR and Chongqing in 2001 and 2010, respectively, were identified as GB3 genotype.

### Yearly and geographic distribution of HRSVB genotypes

**Figure 3:**
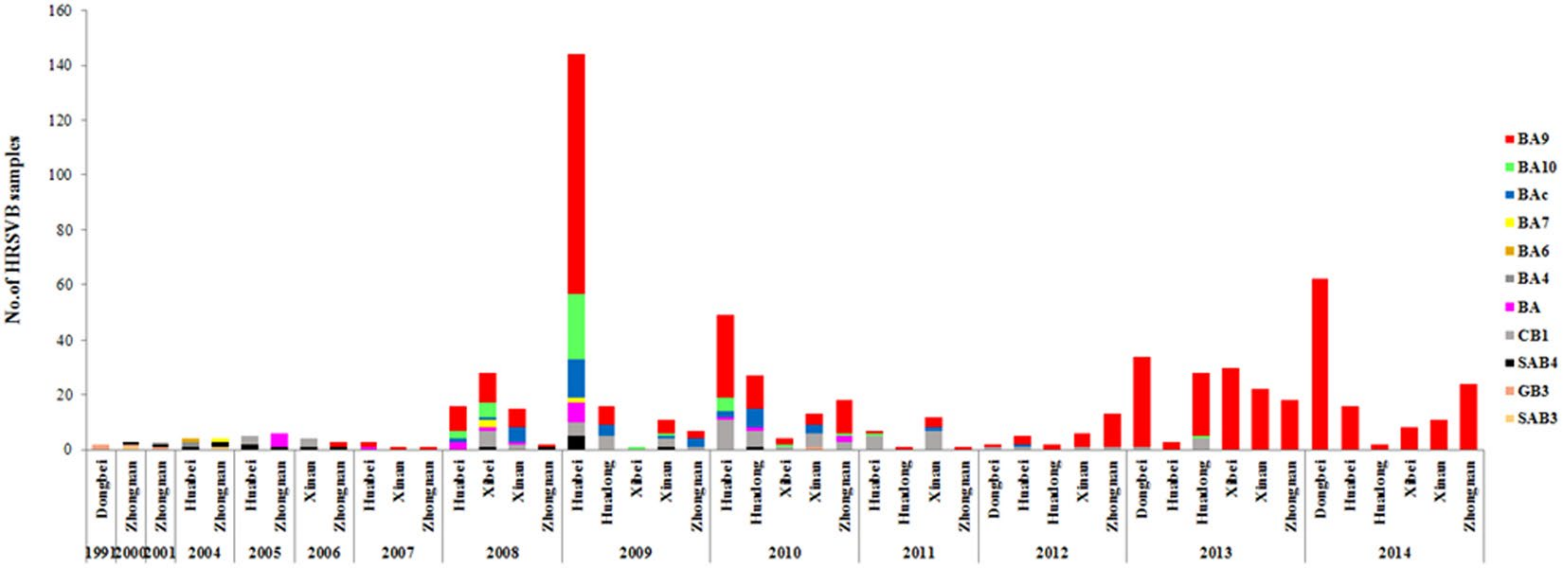
Yearly and geographic distribution of Chinese HRSVB genotypes from 1991 to 2014.

Genotypes SAB and CB1 were the predominant HRSVB genotypes in China before 2007 (Fig. 3). Following that year, the BA genotype became the main HRSVB genotype and circulated with the CB1 genotype simultaneously in China until 2013. The first genotype BA sample identified in this study was collected from Beijing in 2004. From 2008 to 2010, the BA9 genotype was the most prevalent genotype in China, co-circulating with a small number of HRSVB samples of the BA10, BAc, and CB1 genotypes. From 2012 to 2014, the number of the HRSVB sequences that clustered with the BA9 genotype increased substantially, with the prevalence of BA9 reaching almost 100% of HRSVB. In contrast, the BA10 genotype was found circulating intermittently in China in 2008–2011. It was most frequently detected in 2009 and 2010, but the number of BA10 samples gradually decreased in the following years to the level that no BA10 samples were detected in 2014 (Fig. 3).

### Phylogenetic analysis of genotype BA9 and BA10 sequences from China and other parts of the world

**Figure 4:**
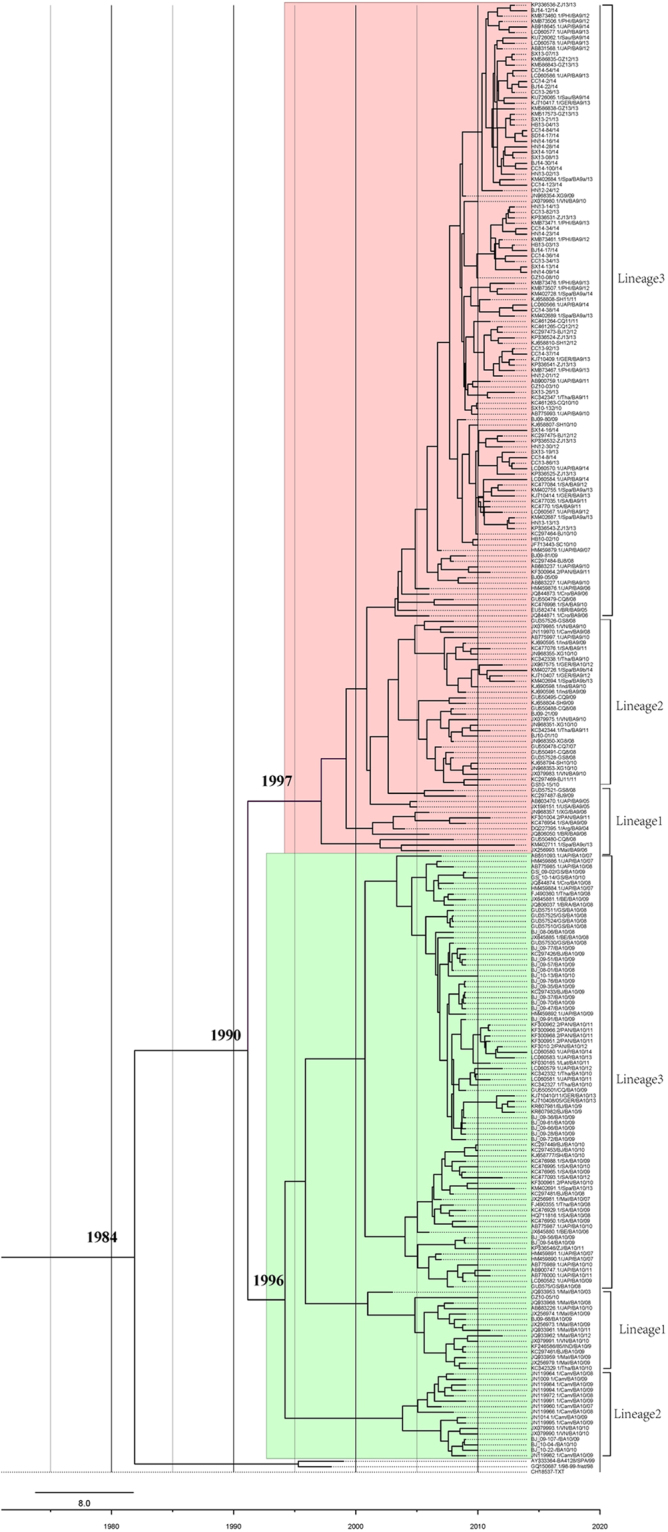
Maximum clade credibility tree of genotypes BA9 and BA10 of HRSVB. Sequences of the BA9 genotype were shaded in pink and those of the BA10 genotype were shaded in green.

The spread of the BA9 and BA10 genotypes has been a worldwide phenomenon in recent years. To study the relationship of the BA9 and BA10 genotypes from China with those from other parts of the world, 92 representative Chinese BA9 sequences were selected based on their yearly and geographic origins, as well as their nucleotide diversities to be analyzed in phylogenetic studies with 64 global BA9 reference sequences downloaded from GenBank. Sequences from the BA9 genotype could be divided into 3 potential lineages, named lineages 1–3 (Fig. 2b). Lineage 1 consisted of sequences from 4 strains from the Huabei, Xinan, Zhongnan and Xibei regions of China collected in 2006, 2008–2009, as well as sequences from Japan, USA, Panama, Argentina, South Africa, Brazil and Croatia between 2004 and 2011. Sequences from the BA9 genotype collected from all regions of China, except the Dongbei region, clustered to lineage 2, together with sequences from other Asian (India, Malaysia, Thailand, Vietnam, Japan, and Cambodia) and European (Spain and Germany) countries from 2009 to 2010. Lineage 3 was comprised of sequences from countries in different parts of the world, including those from Asia (China, Philippines, Vietnam, Japan, Saudi Arabia, Thailand and Malaysia), Europe (Spain, Germany and Croatia), South America and Panama; most of the sequences were collected from 2010–2014 (Figs 2b and 4).

Three potential lineages were also detected when 43 Chinese BA10 sequences were analyzed with 71 global BA10 sequences. Only 3 BA10 sequences collected from Huabei and Zhongnan regions of China in 2009–2010 clustered with BA10 sequences from other Asian countries (Malaysia, Japan, Thailand and Vietnam) in lineage 1. Sequences in lineage 2 were from China, Cambodia, Vietnam and Germany, while sequences in lineage 3, the biggest lineage, were from a broader list of countries including those from Asia, Europe, South Africa and America (Figs 2c and 4).

### Evolutionary rate of the BA9 and BA10 genotypes

**Figure 5:**
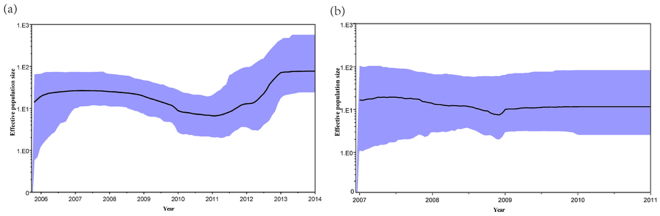
(**a**) Bayesian skyline plot with 92 Chinese BA9 sequences from 2006 to 2014. (**b**) Bayesian skyline plot with 43 Chinese BA10 sequences from 2008 to 2011. The thick solid line is the estimated median, and the grey areas show the upper and lower bounds of the 95% HPD interval.

The evolutionary rate of the BA9 genotype based on a collection of 156 global sequences (92 sequences from China) was 4.53 × 10^−3^ substitutions/site/year, and the time to the most recent common ancestor (TMRCA) was estimated for the year 1997 (Table 2, Fig. 4). In addition, the evolutionary rate of the BA10 genotype based on a collection of 114 global sequences (43 sequences from China) was 4.21 × 10^−3^ substitutions/site/year and the TMRCA was estimated for the year 1996. The evolutionary rates of the BA9 and BA10 genotypes based on Chinese sequences only were similar to those estimated from global sequences (Table 2). Finally, the combined evolutionary rate of the BA9 and BA10 genotypes was estimated to be 4.03 × 10^−3^ substitutions/site/year and they were estimated to diverge from the BA1 genotype around 1990. Bayesian skyline plot (BSP) analysis showed that the effective population size of the BA9 genotype had a slight decrease from 2009 to 2011 and increased from 2011 to 2014 (Fig. 5a). In contrast, the genetic diversity of the viral population of the BA10 genotype remained steady from 2009 to 2011 as the effective population size of the BA10 genotype was constant in this period of time (Fig. 5b).

**Table 2:** Evolutionary rates and TMRCA of genotypes BA9 and BA10.

Genotype	Number of sequences	Evolutionary rate (95% HPD) (substitution/site/year × 10^−3^)	TMRCA(95%HPD) (year)
BA9	156(92CHN)	4.53 (3.4, 5.86)	1997 (1985, 2016)
	92CHN	4.38 (2.81, 6.12)	1996 (1986, 2017)
BA10	114(43CHN)	4.21 (2.94, 5.55)	1996 (1984, 2015)
	43CHN	4.13 (2.01, 6.63)	1997 (1992, 2016)
BA9/BA10	267(130CHN)	4.03 (2.99, 5.09)	1990 (1975, 2015)

CHN: Chinese sequences; HPD: Highest probability density; TMRCA: Time to the most recent common ancestor.

## Discussion

HRSV infection is the main cause of ALRTI in the pediatric population worldwide. Although there were studies that investigated the clinical characteristics, circulation, and/or molecular epidemiology of HRSV infection in some regional areas within China^11,17,22,23,25^, there is almost no large-scale study that examined these topics for the whole country. We recently reported the circulating patterns of HRSVA and HRSVB, and the detailed molecular epidemiology of HRSVA throughout China from 2008 to 2015^18^. The present study provided an extensive epidemiological analysis of HRSVB infection as well as phylogenetic and evolutionary analysis of HRSVB genotypes circulating in 6 representative geographic regions in China from 1991 to 2014.

The first HRSV BA strain with 60-nt duplication in the HVR2 of G protein was isolated in Madrid in 1998^19^, and first reported BA strains isolated from Buenos Aires in 1999^26^. After that, the BA genotype had spread all over the world and evolved into 12 genotypes^19^. Although the number of HRSVB sequences collected before 2008 was relatively small in this study, it is apparent that there was a co-circulation of the BA-genotype with the non-BA genotype. In particular, there were a multiple of major HRSVB genotypes co-circulating in China between 2008 to 2014, including BA9, CB1, BAc and BA10. In Kenya, the genotype BA, especially the BA9 genotype, and the non-BA genotype were also found to have emerged together in the 2013/2014 epidemic season^27^.

The BA9 and BA10 genotypes have spread widely throughout the world in recent years. The BA9 genotype was first identified in Japan in the 2006/2007 epidemic season and it became the predominant genotype in the 2009/2010 epidemic season in Japan. The BA9 genotype also clustered with most of HRSVB strains in Korea from 2008 to 2010, and it was also the main BA genotype that circulated in the Philippines from 2009 to 2011^10,16,28^. In Kenya, the outbreak of HRSV infection was associated predominantly with the genotype BA, especially the BA9 genotype in the 2013/2014 epidemic season^27^. Thus, the BA9 genotype has circulated in Asian countries outside China at least from the 2006 to 2011 epidemic seasons.

The BA9 genotype was first detected in China in 2006 and became the predominant HRSVB genotype circulating in China from 2008 to 2014. The BA10 genotype co-circulated with the BA9 genotype from 2008 to 2011, but no BA10 sequences were detected after 2011. A recent study reported that the detection of the BA9 and BA10 genotypes decreased from 2010 to 2014 in Japan, and predicted both genotypes may decline in Japan in the near future^29^. Our detection of the decline of the BA10 genotype in China was consistent with that reported in Japan, but our study found that genotype BA9, unlike the situation in Japan, gradually increased in prevalence in China between 2010 and 2014. It will be interesting to monitor if the BA10 genotype would reappear in the future or the BA9 genotype would circulate in China for a long period of time.

Phylogenetic analysis revealed that sequences of the BA9 genotype collected from China could be grouped into 3 lineages with sequences collected from other parts of the world. All of these 3 lineages could be detected in countries in Asia (e.g. China, Philippines, Vietnam, Japan, Saudi Arabia, Thailand and Malaysia), Europe (Spain, Brazil, Belgium, Germany and Croatia), South Africa and Panama from 2010 to 2014. For example, the sequences in lineage 3 from Japan^29^, Spain^30^ and Germany^31^ were named as lineage 1, BA9-A and Cl3, respectively; sequences in lineage 2 were named as BA9-B and Cl2 in Spain and Germany, respectively^29–31^. These data suggested that these 3 lineages emerged or circulated widely in Asian and European countries, raising the possibility that BA9 of these lineages were transmitted between China and other countries. Similarly, phylogenetic analysis also showed that the 43 sequences of Chinese BA10 genotype clustered into 3 lineages with sequences collected from other parts of the world. Of interest, lineage 3 of genotype BA10 appeared to have spread widely and Chinese sequences demonstrated high similarity with sequences from Japan^16^, Malaysia^15^, Thailand^32^, and India^33^ suggesting the possibility of transmission of this lineage between China and neighboring countries.

Based on our analysis, the TMRCA of the BA9 or BA10 genotype was estimated to be 1996 or 1997, respectively. However, BA9 and BA10 genotypes were first detected in Japan in 2006 and 2007, indicating that both genotypes remained undetected for 10 years^29^. In addition, the BEAST analysis estimated that the evolutionary rates of the BA9 genotype (4.53 × 10^−3^ substitutions/site/year) and the BA10 genotype (4.21 × 10^−3^ substitutions/site/year) were not significantly different, and these evolutionary rates were similar to that reported by Japan analysis^29^. The BEAST analysis also suggested that the evolution rates of Chinese BA9 and BA10 genotypes were similar to those of the global BA9 and BA10 genotypes. Interestingly, the BSP analysis found that the effective population size of BA9 genotype increased from 2011 till 2014 suggesting that the genetic diversity of Chinese BA9 genotype had increased during this period of time. It remains to be determined if this increase in genetic diversity has contributed to the development of the different BA9 lineages in China. Although it is not clear if the evolution of the BA genotype enhances its spread, the BA genotype has spread widely all over the world during the past 15 years evolving into 12 genotypes^19,27^. It will be important to monitor if the BA9 genotype would continue its spread in the world, or if the BA9 lineage 2 or/and 3 would prevail in China for an extended period of time.

The BAc genotype was only identified in HRSVB from China; 42 BAc sequences clustered away from other genotype sequences with 78% bootstrap value. The BAc genotype was first identified in samples collected in the 2008/2009 and 2009/2010 epidemic seasons^11^. In this study, the BAc genotype was found to be circulating in China from 2008 to 2012, 2 years longer than previously reported. However, no BAc samples were detected in 2013 and 2014. Continued surveillance is necessary to monitor the reappearance of this genotype.

The CB1 genotype was first detected in Beijing in 2009, and the GB5 genotype was first detected in Chongqing in 2010^11,17^. In this study, we confirmed the finding of a previous report that the genotype GB5 samples collected in Chongqing^17^ and the genotype CB1 samples collected in Shanghai^23^, Zhejiang^24^ and Sichuan^25^ were of the same genotype because they clustered together in our phylogenetic analysis. In addition, the sequences identified as CB1 in this study clustered into an independent branch away from any GB genotype reference sequences in the phylogenetic analysis.

There are some limitations of our study due to the difference of sample collection in each year or geographic location that may impact the estimation of the prevalence of HRSVB genotypes in China: the number of HRSVB samples collected before 2008 was small, the number of samples from each province was different each year, and samples were not collected from some provinces every year. In addition, this study was a retrospective study and the source of available samples was limited by the number of samples collected previously.

In conclusion, we found that there was a co-circulation of the BA with the non-BA genotypes in China between 2005 and 2013. The BA9 genotype emerged in 2006 and was the predominant HRSVB genotype circulating in China from 2008 to 2014. The genetic diversity of the BA9 genotype has increased from 2011 to 2014. Hence, a continuous and comprehensive surveillance is needed to monitor the circulation and evolution of the BA and non-BA genotypes to understand the HRSVB transmission in China in the future.

## Methods

### Ethics statement

This study was approved by the second session of the Ethics Review Committee of the National Institute for Viral Disease Control and Prevention of the Center for Disease Control and Prevention (CDC) in China and the methods were performed in accordance with the approved guidelines. There was no human experimentation involved in this study; only nasopharyngeal precipitates were collected from patients with respiratory infections. Written Informed consents for the use of the clinical samples were signed by parents or guardians.

### Specimen collection

Nasopharyngeal precipitates were taken from patients who were hospitalized with acute respiratory illness or outpatients with symptoms of respiratory infections in 1991, 2004 and from 2008 to 2014. This study was approved by 9 sentinel hospitals, from the Dongbei region (Jilin province), Huabei region (Beijing and Hebei provinces), Huadong region (Shandong province and Shanghai), Zhongnan region [Guangdong and Hunan provinces, and Hong Kong special administrative region (SAR)], and Xibei region (Gansu and Shaanxi provinces). Clinical samples were inoculated into HEp-2 cells for virus isolation before storing at −80 °C. Double-channel real-time reverse transcription-polymerase chain reaction (RT-PCR) was conducted to identify the HRSV-positive samples as well as to determine their HRSV subgroups^34^. To confirm the HRSV subgroups of the samples, the sequences were compared with HRSV sequences of known subgroups by the Basic Local Alignment Search Tool (BLAST) program available on the NCBI homepage (http://blast.ncbi.nlmnih.gov.Blast.cgi).

### RNA extraction, DNA amplification, and sequencing

The QIAamp RNA mini kit (QIAGEN, Valencia, CA, USA) was used to extract the total RNA from clinical samples or the isolates according to the manufacturer’s instructions. RT-PCR was performed using Takara one-step RT-PCR kit (TaKaRa Biotechnology, Dalian, China) to amplify the sequence of the region encoding the HVR2 of the G protein (637–968 nt) of HRSVB using forward primer GPB: 5′-AAGATGATTACCATTTTGAAGT-3′, and reverse primer: F1:5′-CAACTCCATTGTTATTTGCC-3′ ^35^. The PCR products were purified using a QIAquick Gel Extraction Kit (QIAGEN), and the purified PCR products were sequenced using an ABI Prism 3710xl DNA Analyzer at Majorbio Co., Ltd. (Beijing, China). The HRSVB sequences were edited using Sequencher software vision 5.0 (Gene Codes, Ann Arbor, MI, USA).

The 365 sequences generated in this study are available in GenBank with accession numbers of DQ289648, DQ289649 and KX892710 to KX893072.

### Additional Chinese HRSVB sequences used in this study

332 Chinese HRSVB sequences that were available in GenBank on July 18, 2016 were downloaded to be analyzed together with the 365 HRSVB sequences generated in this study using the MEGA5.0 software.

### Phylogenetic analysis

The HRSVB sequences were aligned with reference HRSVB sequences using the ClustalW program in the MEGA5.0 software (www.megasoftware.net). The phylogenetic trees were constructed using the neighbor-joining and the maximum composite likelihood methods within MEGA5.0 software, using 1000 replicates of bootstrap probabilities with a cut-off of ≥70% for evaluation of confidence estimates^36,37^.

### Evolutionary rate analysis and estimation of TMRCA

The evolutionary rate and TMRCA were estimated by BEAST (Version 1.8.2)^38^. The jModelTest software (Version 0.1)^39^ was used to calculate the best nucleotide substitution model and the GTR (general time reversible model) + G (gamma) were determined as the best substitution model for BA9 and BA10 genotype sequences. The uncorrelated exponential relaxed clock model was considered to be the best after the Bayes factor test of the strict, uncorrelated log-normal relaxed clock and the uncorrelated exponential relaxed clock. The Markov Chain Monte Carlo chains were run for 100,000,000, and 400,000,000 steps. After 10% burnin, a Maxmum Clade Credibility tree was built by TreeAnnotator (Version 1.8.2) and edited by FigTree (Version 1.4.2). The BSP analysis was conducted using the BEAST1.8.2 package.

## Electronic supplementary material

Supplementary Table1

Supplementary Figure1
