# Supplementary material for: Emergence of BA9 genotype of human respiratory syncytial virus subgroup B in China from 2006 to 2014
Source: Sci Rep. 2017 Dec 1;7:16765. doi: 10.1038/s41598-017-17055-0 (PMC5711796; doi:10.1038/s41598-017-17055-0)
Supplement: Supplementary file 1 — Supplementary Table1 [file 41598_2017_17055_MOESM1_ESM.pdf]

Emergence of BA9 genotype of human respiratory syncytial virus subgroup B in China from 2006 to 2014

Jinhua Song[1], Huiling Wang[1], Jing Shi[1,2], Aili Cui[1], Yanzhi Huang[3], Liwei Sun[3], Xingyu Xiang[4], Chaofeng Ma[5], Pengbo Yu[6], Zifeng Yang[7], Qi Li[8], Teresa I. Ng[9], Yan Zhang[1#] , Rongbo Zhang[10#], Wenbo Xu[1,10#]

[1]WHO WPRO Regional Reference Measles/Rubella Laboratory and Key Laboratory of Medical Virology, Ministry of Health, National Institute for Viral Disease Control and Prevention, China Center for Disease Control and Prevention, Beijing, People's Republic of China.[2] Lu Juan Community Health Center of Daxing region, Beijing, People's Republic of China. [3]Jilin Children's Medical Center, Children's Hospital of Changchun, Changchun, People's Republic of China. [4]Hunan Provincial Centers for Disease Control and Prevention, Changsha, People's Republic of China. [5]Xian Center for Disease Control and Prevention, Xian, People's Republic of China. [6]Shaanxi Provincial Centers for Disease Control and Prevention, Xian, People's Republic of China. [7]State Key Laboratory of Respiratory Disease, National Clinical Research Center for Respiratory Disease, First Affiliated Hospital of Guangzhou Medical University, Guangzhou, People's Republic of China. [8]Hebei Provincial Centers for Disease Control and Prevention, Shijiazhuang, People's Republic of China. [9]AbbVie, Inc., North Chicago, IL, USA. [10]Medical College, Anhui University of Science & Technology, Huainan, People's Republic of China.

[#]corresponding authors:

Yan Zhang, E-mail address: zhangyan9876543@163.com，

Rongbo Zhang, E-mail address: lory456@126.com,

Wenbo Xu, E-mail address: wenbo_xu1@aliyun.com.

Table 1. Additional Chinese HRSVB sequences download from GenBank.

GenBank No.	Subgroup	Provinces/Cities	region
KM517573	B	Guangdong	Zhongnan
KP336523	B	Zhejiang	Huadong
KP336524	B	Zhejiang	Huadong
KP336525	B	Zhejiang	Huadong
KP336526	B	Zhejiang	Huadong
KP336527	B	Zhejiang	Huadong
KP336528	B	Zhejiang	Huadong
KP336529	B	Zhejiang	Huadong
KP336530	B	Zhejiang	Huadong
KP336531	B	Zhejiang	Huadong
KP336532	B	Zhejiang	Huadong
KP336533	B	Zhejiang	Huadong
KP336534	B	Zhejiang	Huadong
KP336535	B	Zhejiang	Huadong
KP336536	B	Zhejiang	Huadong
KP336537	B	Zhejiang	Huadong
KP336538	B	Zhejiang	Huadong
KP336539	B	Zhejiang	Huadong
KP336540	B	Zhejiang	Huadong
KP336541	B	Zhejiang	Huadong
KP336542	B	Zhejiang	Huadong
KP336543	B	Zhejiang	Huadong
KP336544	B	Zhejiang	Huadong
KP336545	B	Zhejiang	Huadong
KP336546	B	Zhejiang	Huadong
GU357503	B	Gansu	Xibei
GU357504	B	Gansu	Xibei

GU357505	B	Gansu	Xibei
GU357508	B	Gansu	Xibei
GU357509	B	Gansu	Xibei
GU357510	B	Gansu	Xibei
GU357511	B	Gansu	Xibei
GU357512	B	Gansu	Xibei
GU357514	B	Gansu	Xibei
GU357515	B	Gansu	Xibei
GU357516	B	Gansu	Xibei
GU357520	B	Gansu	Xibei
GU357521	B	Gansu	Xibei
GU357523	B	Gansu	Xibei
GU357524	B	Gansu	Xibei
GU357525	B	Gansu	Xibei
GU357526	B	Gansu	Xibei
GU357527	B	Gansu	Xibei
GU357528	B	Gansu	Xibei
GU357529	B	Gansu	Xibei
GU357530	B	Gansu	Xibei
GU550479	B	Chongqing	Xinan
GU550480	B	Chongqing	Xinan
GU550482	B	Chongqing	Xinan
GU550483	B	Chongqing	Xinan
GU550484	B	Chongqing	Xinan
GU550485	B	Chongqing	Xinan
GU550486	B	Chongqing	Xinan
GU550487	B	Chongqing	Xinan
GU550488	B	Chongqing	Xinan
GU550490	B	Chongqing	Xinan
GU550491	B	Chongqing	Xinan
GU550492	B	Chongqing	Xinan
GU550493	B	Chongqing	Xinan
GU550494	B	Chongqing	Xinan
GU550495	B	Chongqing	Xinan
GU550496	B	Chongqing	Xinan

GU550498	B	Chongqing	Xinan
GU550501	B	Chongqing	Xinan
GU550502	B	Chongqing	Xinan
GU550503	B	Chongqing	Xinan
JF713439	B	Sichuan	Xinan
JF713440	B	Sichuan	Xinan
JF713442	B	Sichuan	Xinan
JF713443	B	Sichuan	Xinan
JN968345	B	Hong Kong	Zhongnan
JN968346	B	Hong Kong	Zhongnan
JN968350	B	Hong Kong	Zhongnan
JN968351	B	Hong Kong	Zhongnan
JN968352	B	Hong Kong	Zhongnan
JN968353	B	Hong Kong	Zhongnan
JN968354	B	Hong Kong	Zhongnan
JN968355	B	Hong Kong	Zhongnan
JN968356	B	Hong Kong	Zhongnan
JN968358	B	Hong Kong	Zhongnan
JN968359	B	Hong Kong	Zhongnan
JN968362	B	Hong Kong	Zhongnan
JN968363	B	Hong Kong	Zhongnan
KC297422	B	Beijing	Huabei
KC297423	B	Beijing	Huabei
KC297424	B	Beijing	Huabei
KC297425	B	Beijing	Huabei
KC297426	B	Beijing	Huabei
KC297429	B	Beijing	Huabei
KC297431	B	Beijing	Huabei
KC297433	B	Beijing	Huabei
KC297434	B	Beijing	Huabei
KC297435	B	Beijing	Huabei
KC297436	B	Beijing	Huabei
KC297437	B	Beijing	Huabei
KC297438	B	Beijing	Huabei
KC297439	B	Beijing	Huabei

KC297440	B	Beijing	Huabei
KC297441	B	Beijing	Huabei
KC297442	B	Beijing	Huabei
KC297443	B	Beijing	Huabei
KC297444	B	Beijing	Huabei
KC297445	B	Beijing	Huabei
KC297447	B	Beijing	Huabei
KC297448	B	Beijing	Huabei
KC297449	B	Beijing	Huabei
KC297452	B	Beijing	Huabei
KC297453	B	Beijing	Huabei
KC297455	B	Beijing	Huabei
KC297456	B	Beijing	Huabei
KC297457	B	Beijing	Huabei
KC297458	B	Beijing	Huabei
KC297459	B	Beijing	Huabei
KC297460	B	Beijing	Huabei
KC297461	B	Beijing	Huabei
KC297463	B	Beijing	Huabei
KC297464	B	Beijing	Huabei
KC297465	B	Beijing	Huabei
KC297467	B	Beijing	Huabei
KC297469	B	Beijing	Huabei
KC297473	B	Beijing	Huabei
KC297475	B	Beijing	Huabei
KC297476	B	Beijing	Huabei
KC297477	B	Beijing	Huabei
KC297478	B	Beijing	Huabei
KC297479	B	Beijing	Huabei
KC297480	B	Beijing	Huabei
KC297481	B	Beijing	Huabei
KC297482	B	Beijing	Huabei
KC297483	B	Beijing	Huabei
KC297484	B	Beijing	Huabei
KC297485	B	Beijing	Huabei

KC297486	B	Beijing	Huabei
KC297487	B	Beijing	Huabei
KC297488	B	Beijing	Huabei
KC297489	B	Beijing	Huabei
KC297490	B	Beijing	Huabei
KC297491	B	Beijing	Huabei
KC297492	B	Beijing	Huabei
KC297493	B	Beijing	Huabei
KC461262	B	Chongqing	Xinan
KC461263	B	Chongqing	Xinan
KC461264	B	Chongqing	Xinan
KC461265	B	Chongqing	Xinan
KC461266	B	Chongqing	Xinan
KC461267	B	Chongqing	Xinan
KM586835	B	Guangdong	Zhongnan
KM586836	B	Guangdong	Zhongnan
KM586838	B	Guangdong	Zhongnan
KM586840	B	Guangdong	Zhongnan
KM586843	B	Guangdong	Zhongnan
KM586844	B	Guangdong	Zhongnan
KR607978	B	Beijing	Huabei
KR607979	B	Beijing	Huabei
KR607980	B	Beijing	Huabei
KR607981	B	Beijing	Huabei
KR607982	B	Beijing	Huabei
KR607983	B	Beijing	Huabei
KR607984	B	Beijing	Huabei
KJ658777	B	Shanghai	Huadong
KJ658778	B	Shanghai	Huadong
KJ658779	B	Shanghai	Huadong
KJ658780	B	Shanghai	Huadong
KJ658781	B	Shanghai	Huadong
KJ658782	B	Shanghai	Huadong
KJ658783	B	Shanghai	Huadong
KJ658784	B	Shanghai	Huadong

KJ658785	B	Shanghai	Huadong
KJ658786	B	Shanghai	Huadong
KJ658787	B	Shanghai	Huadong
KJ658788	B	Shanghai	Huadong
KJ658789	B	Shanghai	Huadong
KJ658790	B	Shanghai	Huadong
KJ658791	B	Shanghai	Huadong
KJ658792	B	Shanghai	Huadong
KJ658793	B	Shanghai	Huadong
KJ658794	B	Shanghai	Huadong
KJ658795	B	Shanghai	Huadong
KJ658796	B	Shanghai	Huadong
KJ658797	B	Shanghai	Huadong
KJ658798	B	Shanghai	Huadong
KJ658799	B	Shanghai	Huadong
KJ658800	B	Shanghai	Huadong
KJ658801	B	Shanghai	Huadong
KJ658802	B	Shanghai	Huadong
KJ658803	B	Shanghai	Huadong
KJ658804	B	Shanghai	Huadong
KJ658805	B	Shanghai	Huadong
KJ658806	B	Shanghai	Huadong
KJ658807	B	Shanghai	Huadong
KJ658808	B	Shanghai	Huadong
KJ658809	B	Shanghai	Huadong
KJ658810	B	Shanghai	Huadong
KJ658764	B	Shanghai	Huadong
KJ658765	B	Shanghai	Huadong
KJ658766	B	Shanghai	Huadong
KJ658767	B	Shanghai	Huadong
KJ658768	B	Shanghai	Huadong
KJ658769	B	Shanghai	Huadong
KJ658770	B	Shanghai	Huadong
KJ658771	B	Shanghai	Huadong
KJ658772	B	Shanghai	Huadong

KJ658773	B	Shanghai	Huadong
KJ658774	B	Shanghai	Huadong
KJ658775	B	Shanghai	Huadong
GU550499	B	Chongqing	Xinan
GU550500	B	Chongqing	Xinan
GU550497	B	Chongqing	Xinan
GU550489	B	Chongqing	Xinan
GU550481	B	Chongqing	Xinan
JF713441	B	Sichuan	Xinan
GU357517	B	Gansu	Xibei
GU357518	B	Gansu	Xibei
GU357522	B	Gansu	Xibei
GU357519	B	Gansu	Xibei
GU357506	B	Gansu	Xibei
GU357507	B	Gansu	Xibei
KP336508	B	Zhejiang	Huadong
KP336509	B	Zhejiang	Huadong
KP336510	B	Zhejiang	Huadong
KP336511	B	Zhejiang	Huadong
GU357513	B	Gansu	Xibei
KC297450	B	Beijing	Huabei
KC297427	B	Beijing	Huabei
KC297428	B	Beijing	Huabei
KC461288	B	Chongqing	Xinan
KC461289	B	Chongqing	Xinan
KC461290	B	Chongqing	Xinan
KC461291	B	Chongqing	Xinan
KC461292	B	Chongqing	Xinan
KC461293	B	Chongqing	Xinan
KC461294	B	Chongqing	Xinan
KC461295	B	Chongqing	Xinan
KC297446	B	Beijing	Huabei
KC297454	B	Beijing	Huabei
KC297462	B	Beijing	Huabei
KC297451	B	Beijing	Huabei

KC297466	B	Beijing	Huabei
KC297468	B	Beijing	Huabei
KC297470	B	Beijing	Huabei
KC297471	B	Beijing	Huabei
KC297472	B	Beijing	Huabei
KC297474	B	Beijing	Huabei
KC297432	B	Beijing	Huabei
KC297430	B	Beijing	Huabei
JF713444	B	Sichuan	Xinan
JF713445	B	Sichuan	Xinan
JF713446	B	Sichuan	Xinan
JN968293	B	Hong Kong	Zhongnan
JN968295	B	Hong Kong	Zhongnan
JN968343	B	Hong Kong	Zhongnan
DQ270227	B	Beijing	Huabei
DQ270228	B	Beijing	Huabei
DQ270229	B	Beijing	Huabei
DQ270230	B	Beijing	Huabei
DQ270231	B	Beijing	Huabei
DQ270232	B	Beijing	Huabei
DQ270233	B	Beijing	Huabei
GU550478	B	Chongqing	Xinan
GU550477	B	Chongqing	Xinan
GU550476	B	Chongqing	Xinan
GU550475	B	Chongqing	Xinan
GU550474	B	Chongqing	Xinan
JN968366	B	Hong Kong	Zhongnan
JN968365	B	Hong Kong	Zhongnan
JN968364	B	Hong Kong	Zhongnan
JN968361	B	Hong Kong	Zhongnan
JN968360	B	Hong Kong	Zhongnan
JN968357	B	Hong Kong	Zhongnan
JN968349	B	Hong Kong	Zhongnan
JN968348	B	Hong Kong	Zhongnan
JN968347	B	Hong Kong	Zhongnan

JN968344	B	Hong Kong	Zhongnan
JN968342	B	Hong Kong	Zhongnan
JN968297	B	Hong Kong	Zhongnan
JN968296	B	Hong Kong	Zhongnan
JN968294	B	Hong Kong	Zhongnan
JN968292	B	Hong Kong	Zhongnan
JN968291	B	Hong Kong	Zhongnan
JN968290	B	Hong Kong	Zhongnan
JN968289	B	Hong Kong	Zhongnan
JN968288	B	Hong Kong	Zhongnan
JN968287	B	Hong Kong	Zhongnan
KT765102	B	Sichuan	Xinan
KT765101	B	Sichuan	Xinan
KT765100	B	Sichuan	Xinan
KT765099	B	Sichuan	Xinan
KT765098	B	Sichuan	Xinan
KT765097	B	Sichuan	Xinan
KT765096	B	Sichuan	Xinan
KT765095	B	Sichuan	Xinan
KT765094	B	Sichuan	Xinan
KT765093	B	Sichuan	Xinan
KT765092	B	Sichuan	Xinan
KT765091	B	Sichuan	Xinan
KT765090	B	Sichuan	Xinan
KT765089	B	Sichuan	Xinan
KT765088	B	Sichuan	Xinan
KT765087	B	Sichuan	Xinan
KT765086	B	Sichuan	Xinan
KT765085	B	Sichuan	Xinan
KT765084	B	Sichuan	Xinan
KT765083	B	Sichuan	Xinan
KT765082	B	Sichuan	Xinan
KT765081	B	Sichuan	Xinan
KT765080	B	Sichuan	Xinan
KT765079	B	Sichuan	Xinan

KT765078	B	Sichuan	Xinan
KT765077	B	Sichuan	Xinan
KT765076	B	Sichuan	Xinan
KT765075	B	Sichuan	Xinan
KT765074	B	Sichuan	Xinan
KT765073	B	Sichuan	Xinan
KT765072	B	Sichuan	Xinan
KT765071	B	Sichuan	Xinan
KT765070	B	Sichuan	Xinan
KT765069	B	Sichuan	Xinan
KT765068	B	Sichuan	Xinan
KT765067	B	Sichuan	Xinan
KT765066	B	Sichuan	Xinan
KT765065	B	Sichuan	Xinan
KT765064	B	Sichuan	Xinan
KT765063	B	Sichuan	Xinan
KT765062	B	Sichuan	Xinan
KT765061	B	Sichuan	Xinan
KT765060	B	Sichuan	Xinan
KT781382	B	Hunan	Zhongnan
KT781358	B	Hunan	Zhongnan
KT781373	B	Hunan	Zhongnan
KT781403	B	Hunan	Zhongnan
KT781397	B	Hunan	Zhongnan
KT781359	B	Hunan	Zhongnan
