# Supplementary material for: Emergence of BA9 genotype of human respiratory syncytial virus subgroup B in China from 2006 to 2014
Source: Sci Rep. 2017 Dec 1;7:16765. doi: 10.1038/s41598-017-17055-0 (PMC5711796; doi:10.1038/s41598-017-17055-0)
Supplement: Supplementary file 2 — Supplementary Figure1 [file 41598_2017_17055_MOESM2_ESM.pdf]

Emergence of BA9 genotype of human respiratory syncytial virus subgroup B in China from 2006 to 2014

Jinhua Song[1], Huiling Wang[1], Jing Shi[1,2], Aili Cui[1], Yanzhi Huang[3], Liwei Sun[3], Xingyu Xiang[4], Chaofeng Ma[5], Pengbo Yu[6], Zifeng Yang[7], Qi Li[8], Teresa I. Ng[9], Yan Zhang[1#], Rongbo Zhang[10#], Wenbo Xu[1,10#]

[1]WHO WPRO Regional Reference Measles/Rubella Laboratory and Key Laboratory of Medical Virology, Ministry of Health, National Institute for Viral Disease Control and Prevention, China Center for Disease Control and Prevention, Beijing, People's Republic of China.[2] Lu Juan Community Health Center of Daxing region, Beijing, People's Republic of China. [3]Jilin Children's Medical Center, Children's Hospital of Changchun, Changchun, People's Republic of China. [4]Hunan Provincial Centers for Disease Control and Prevention, Changsha, People's Republic of China. [5]Xian Center for Disease Control and Prevention, Xian, People's Republic of China. [6]Shaanxi Provincial Centers for Disease Control and Prevention, Xian, People's Republic of China. [7]State Key Laboratory of Respiratory Disease, National Clinical Research Center for Respiratory Disease, First Affiliated Hospital of Guangzhou Medical University, Guangzhou, People's Republic of China. [8]Hebei Provincial Centers for Disease Control and Prevention, Shijiazhuang, People's Republic of China. [9]AbbVie, Inc., North Chicago, IL, USA. [10]Medical College, Anhui University of Science & Technology, Huainan, People's Republic of China.

[#]corresponding authors:

Yan Zhang, E-mail address: zhangyan9876543@163.com，

Rongbo Zhang, E-mail address: lory456@126.com,

Wenbo Xu, E-mail address: wenbo_xu1@aliyun.com.

Supplementary Figure 1. Phylogenetic tree of 697 Chinese HRSVB sequences from 1991 to 2014 with reference sequences from Genbank.

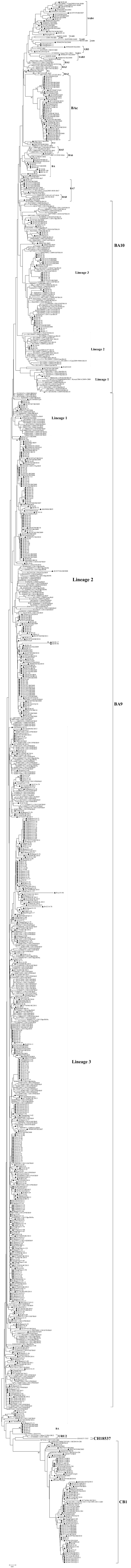

BA10
Lineage 3
Lineage 2
Lineage 1
Lineage 1
Lineage 2
Lineage 3
BA9
CH18537
CB1
